# Supplementary material for: High speed underwater hydrogel robots with programmable motions powered by light
Source: Nat Commun. 2023 Nov 23;14:7672. doi: 10.1038/s41467-023-43576-6 (PMC10667353; doi:10.1038/s41467-023-43576-6)
Supplement: Supplementary file 1 — Supplementary information [file 41467_2023_43576_MOESM1_ESM.pdf]

Supplementary Information

High speed hydrogel underwater robots with programmable motions powered by light

Chujun Ni[1], Di Chen[2], Xin Wen[1], Binjie Jin[1], Yi He[1], Tao Xie[1,2], Qian Zhao[1,2]*

[1] State Key Laboratory of Chemical Engineering, College of Chemical and Biological Engineering, Zhejiang University, Hangzhou 310027, China.

2 Ningbo innovation center, Zhejiang University, Ningbo 315100, China.

*Correspondence to: qianzhao@zju.edu.cn

Supplementary Note

Kinetics estimation of mass and heat transportation

1. Mass transportation

Mass transportation kinetics of bilayer structure is simplified as a one-dimensional problem. The time for the hydrogel to reach fully swelling/deswelling is defined as the mass transportation time t_{mass}. According to the equation reported[1], it can be calculated as $t_{mass}=\frac{d^2}{\pi^2 D_m}$, where D_m represents mass diffusion coefficient and d represents the thickness of the hydrogel. The diffusion coefficient of PNIPAM hydrogel during swelling/deswelling is around 10^{-7} cm^2 • s^{-1} [2-4]. Therefore, t_{mass} varies from 10^6 s • cm^{-2} × d^2.

2. Heat transportation

Heat transportation kinetics is estimated by unsteady heat conduction in solid model. Specifically, the hydrogel is considered homogenous in T_0 (25 °C) initially. Then its surface temperature is suddenly increased to and afterwards maintained at T_1 (60 °C). The time when the center of the gel reaches LCST (namely, T_2 = 32 °C) is defined as the heat transportation time t_{heat}.

According to the equation reported[5], the time for mass transportation $t_{heat}=\frac{d^2}{8D_h}(\ln\frac{T_1-T_0}{T_2-T_1})$, where d and D_h represent the thickness of the hydrogel and the thermal conduction coefficient respectively.

D_h can be calculated as $D_h = \frac{k}{\rho\, c_p}$, where k, ρ and c_p correspond to heat conductivity, density, and heat capacity of the hydrogel, respectively. As reported[6-8], the parameter values are $k \approx 0.5$ W • m^{-1} • K^{-1}, $\rho \approx 1.13$ g • cm^{-3}, $c_p \approx 3$~5 J • g^{-1} • K^{-1}, and D_h is around 10^{-3} cm^2 • s^{-1}. Thereafter, t_{heat} can be calculated as 10^2 s • cm^{-2} × d^2.

When the sample thickness varies from 0.5 mm to 3 mm, t_{heat} varies from 10^{-1} ~10^{1} s, which is much faster than t_{mass} varying from 10^{3} to 10^{5} s.

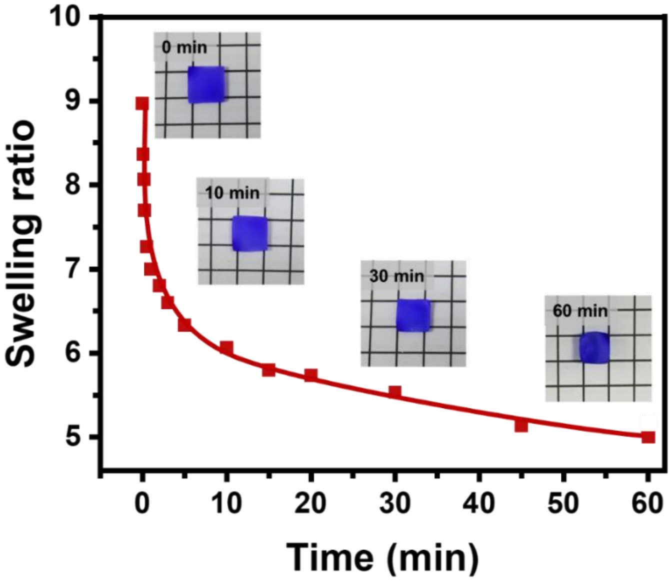

Supplementary Fig. 1 Deswelling kinetics and photographs of the macroscopic isotropic volume shrinkage of an as-synthesized PNIPAM hydrogel.

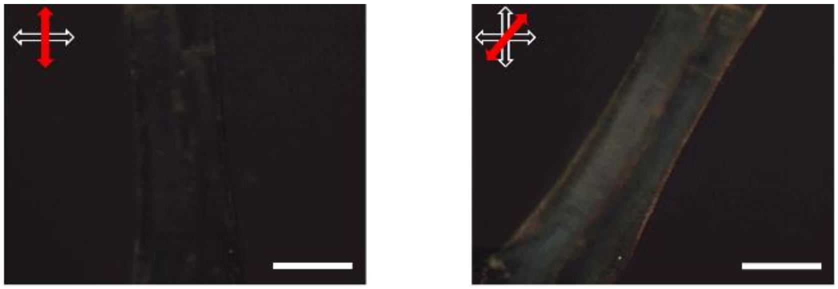

Supplementary Fig. 2 Polarized optical microscopy photographs of a programmable hydrogel sample. The strain was 100%, and the irradiation time was 5 min. The scale bars are 100 μm.

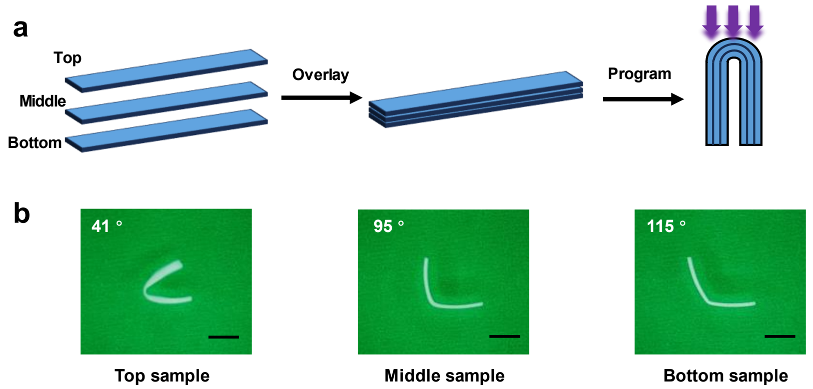

Supplementary Fig. 3 Programming of laminated samples. a, Scheme of the programming process. **b,** Photographs of the samples after programming from top to bottom. Scale bars are 0.5 cm.

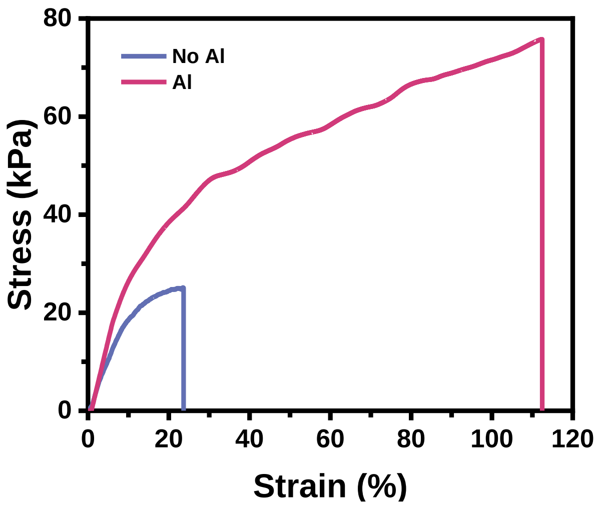

Supplementary Fig. 4 Tensile tests of PNIPAM hydrogels with or without Al ion coupling.

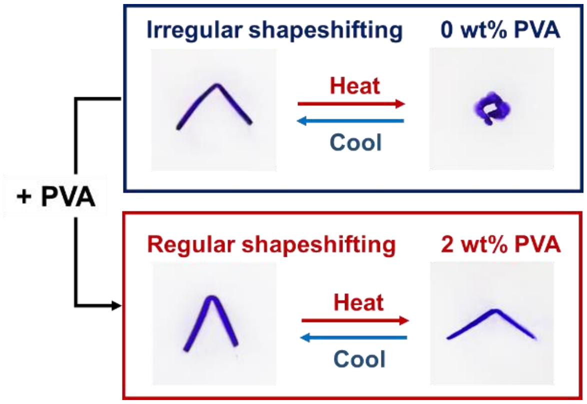

Supplementary Fig. 5 Comparison of shapeshifting behavior of the hydrogels containing PVA or not.

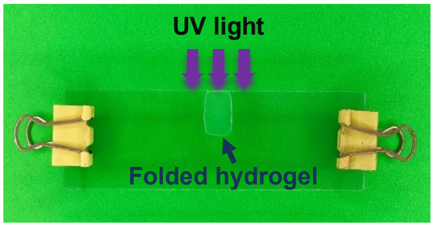

Supplementary Fig. 6 Illustration showing the fixing of the bending deformation in Fig. 2a.

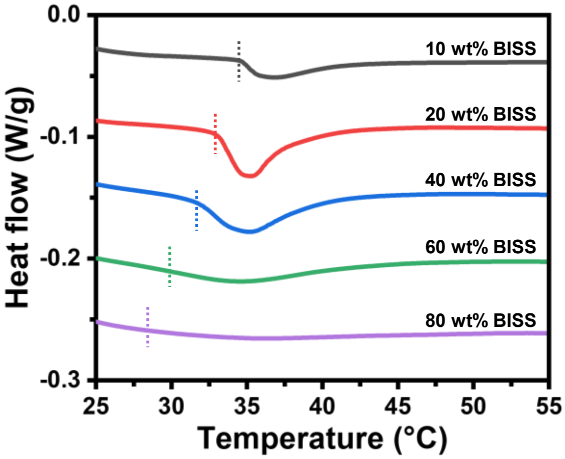

Supplementary Fig. 7 DSC curves of PNIPAM hydrogels with different BISS contents.

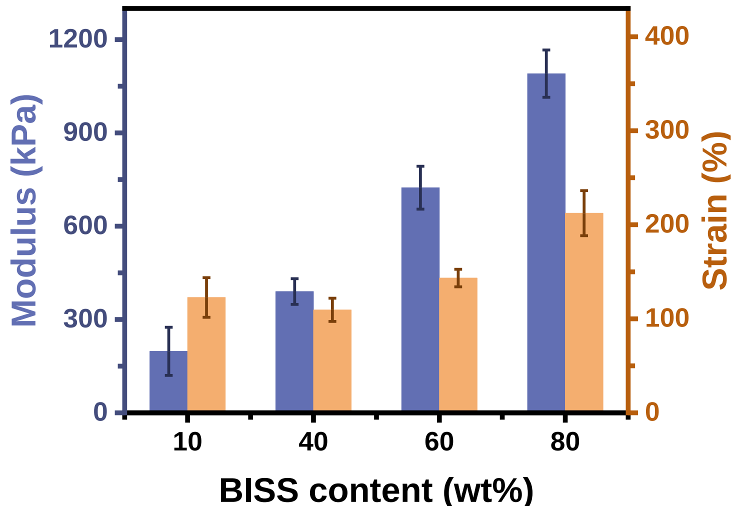

Supplementary Fig. 8 Mechanical properties of hydrogel samples with different BISS contents. Error bars correspond to s.d. (n = 5).

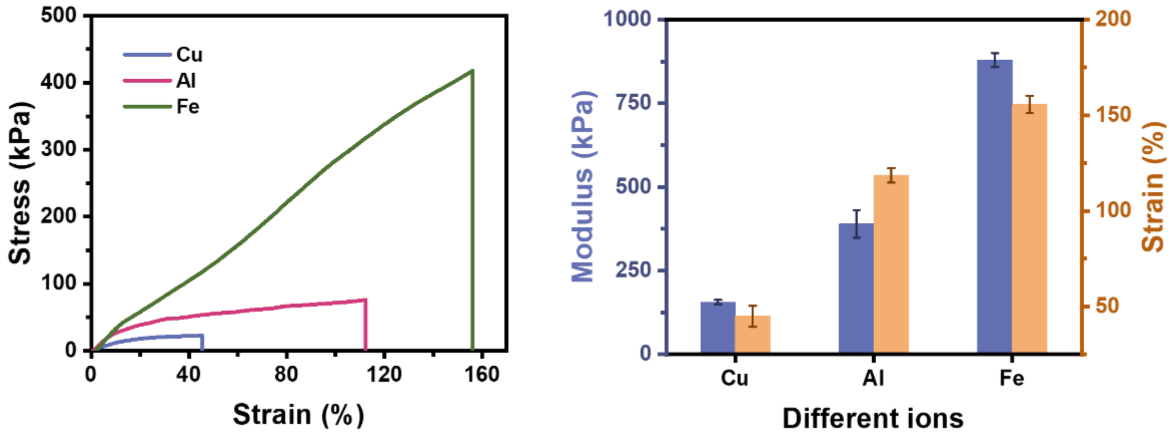

Supplementary Fig. 9 Mechanical properties of hydrogels with different ionic crosslinking. Error bars correspond to s.d. (n = 5).

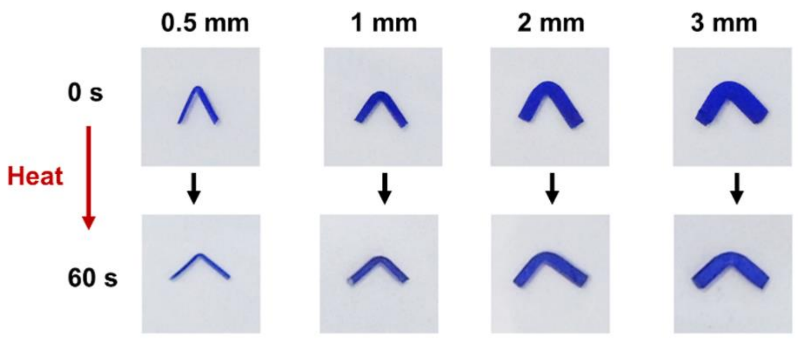

Supplementary Fig. 10 Macroscopic actuation of hydrogel samples with different thickness in 60 s.

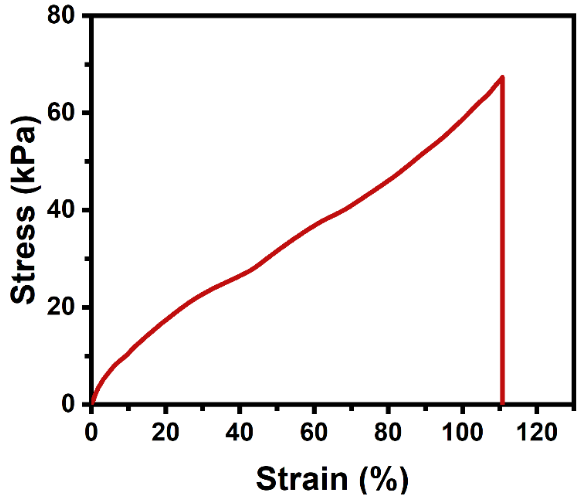

Supplementary Fig. 11 Mechanical property of carbon-doped hydrogel.

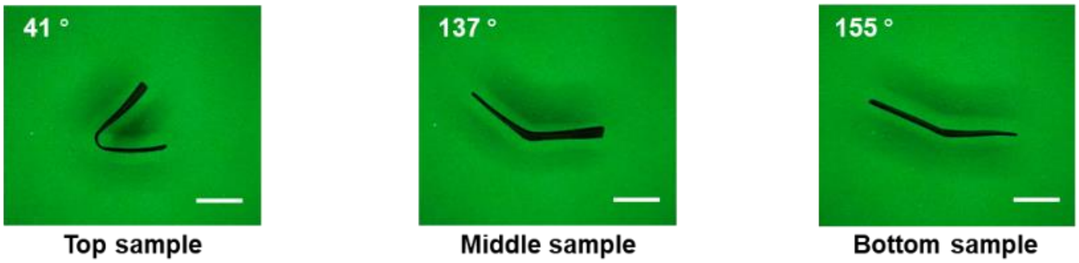

Supplementary Fig. 12 Gradient distribution of the carbon-dopped samples. The experiment method is the same as that in Supplementary Fig. 3.

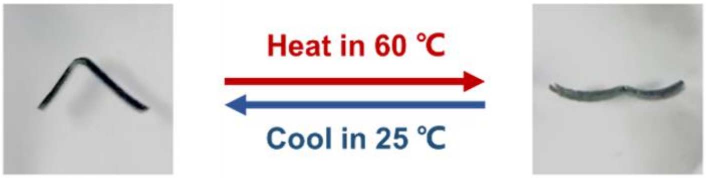

Supplementary Fig. 13 Actuation performance of a carbon-dopped hydrogel sample. The shape retention was 53.2±2.2 % and the actuation angle was 74.7±4.2°, which were calculated from five parallel samples.

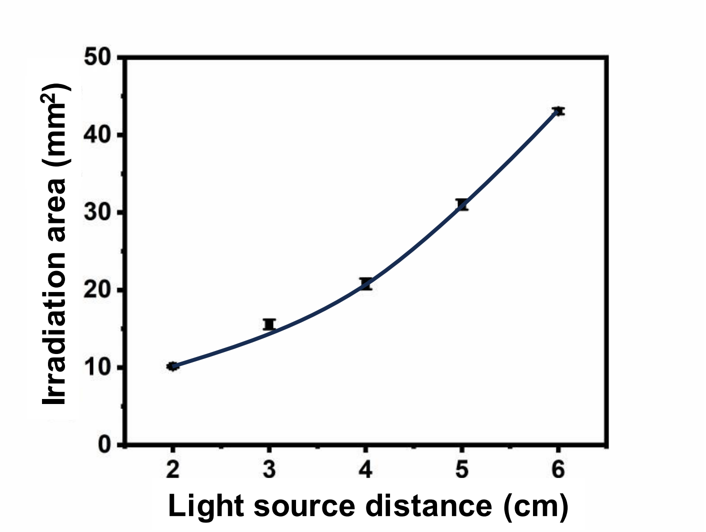

Supplementary Fig. 14 NIR-irradiation area with various distances between the light source and the sample. The irradiation area is calculated from the NIR spot area of three parallel tests.

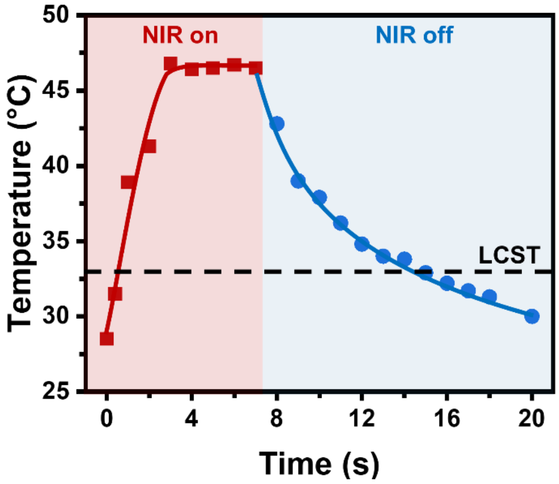

Supplementary Fig. 15 Photo thermal effect of the carbon-doped hydrogel. The temperature increased as soon as turning on NIR light and recovered upon turning off NIR. The process was carried out under ambient water throughout the process.

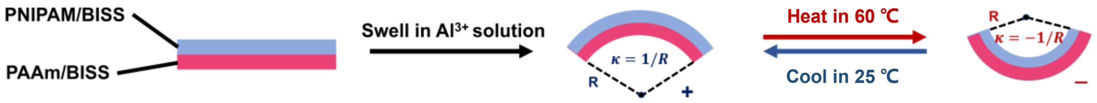

Supplementary Fig. 16 The synthesis of conventional thermo-responsive bilayer hydrogel.

Supplementary References

1. Doi, M. Gel dynamics. *J. Phys. Soc. Jpn.* **78**, 052001 (2009).
2. László, K., Kosik, K. & Geissler, E. High-sensitivity isothermal and scanning microcalorimetry in PNIPAM hydrogels around the volume phase transition. *Macromolecules* **37**, 10067-10072 (2004).
3. Fu, G. & Soboyejo, W. O. Investigation of swellable poly (*N*-isopropylacrylamide) based hydrogels for drug delivery. *Mater. Sci. Eng. C* **31**, 1084-1090 (2011).
4. Lue, S. J., Chen, C.-H. & Shih, C.-M. Tuning of lower critical solution temperature (LCST) of poly(*N*-isopropylacrylamide-co-acrylic acid) hydrogels. *J. Macromol. Sci. Part B-Phys.* **50**, 563-579 (2011).
5. Yu, C. et al. Hydrogels as dynamic memory with forgetting ability. *Proc. Natl. Acad. Sci. U.S.A.* **117**, 18962-18968 (2020).
6. Xu, S., Cai, S. & Liu, Z. Thermal conductivity of polyacrylamide hydrogels at the nanoscale. *ACS Appl. Mater. Interfaces* **10**, 36352-36360 (2018).
7. Dubovik, A. S., Kuznetsov, D. V., Grinberg, N. V., A. Grosberg, Yu. & Tanaka, T. Studies of the thermal volume transition of poly(*N*-isopropylacrylamide) hydrogels by high-sensitivity differential scanning microcalorimetry. 2. thermodynamic functions. *Macromolecules* **33**, 8685-8692 (2000).
8. Tang, N. et al. Thermal transport in soft PAAm hydrogels. *Polymers* **9**, 688 (2017).
