# Supplementary material for: High speed underwater hydrogel robots with programmable motions powered by light
Source: Nat Commun. 2023 Nov 23;14:7672. doi: 10.1038/s41467-023-43576-6 (PMC10667353; doi:10.1038/s41467-023-43576-6)
Supplement: Supplementary file 3 — Description of additional supplementary files [file 41467_2023_43576_MOESM3_ESM.pdf]

Description of additional supplementary files

Supplementary Movie 1 Reversible bloom and close of a hydrogel flower

Supplementary Movie 2 Comparison of response speed between a traditional hydrogel actuator and the programmed actuator

Supplementary Movie 3 Photo thermal actuation frequency of a carbon-dopped hydrogel sample

Supplementary Movie 4 A high-speed hydrogel swimmer

Supplementary Movie 5 A fast hydrogel crawler

Supplementary Movie 6 A hydrogel rotator with fast response
